# Factors Associated with Research Literacy in University Students: A Systematic Literature Review 2015-2025

**DOI:** 10.12688/f1000research.179559.2

**Published:** 2026-10-17

**Authors:** ANA ELIZABETH PAREDES MORALES, Marilú Trinidad Flores Lezama, Rolando Rosmer Coronado Orrillo,, José Luis Núñez Pesantes, Jacqueline Yovani Palacios Rodríguez, Carlos Alberto Barbarán Villegas

**Affiliations:** 1 La Libertad, Universidad Cesar Vallejo, Trujillo, La Libertad, 13871, Peru

**Keywords:** Research literacy, Information literacy, Higher education, Systematic review, University students

## Abstract

This systematic literature review analyses factors associated with research literacy among university students based on 50 studies published between 1 January 2015 and 31 December 2025. Following PRISMA guidelines, studies were selected from Scopus, Web of Science, and Science Direct databases. The findings reveal research literacy as a multidimensional competency operating through three interconnected dimensions — personal (63%), pedagogical (23%) and institutional (14%), computed over the 35 studies with extractable quantitative data —. Personal factors showed the strongest statistical associations, with information literacy (d = 0.82) and research self-efficacy (d = 0.76) yielding the largest pooled effect sizes. Pedagogical factors, though less represented, were associated with the largest effect sizes, particularly library-faculty collaboration (d = 0.94) and structured instruction (d = 0.78). Institutional factors, including frameworks and technological resources, were consistently associated with larger estimates for personal and pedagogical variables, a pattern compatible with — but not demonstrative of — an amplifying role. The review identified a paradigmatic shift from traditional information literacy toward critical artificial intelligence literacy, with a 410% growth in AI-related studies between 2023–2025. Bibliometric analysis revealed geographical concentration in Europe and Asia (50%), highlighting underrepresentation of Global South perspectives. Methodological limitations include predominance of correlational designs (36%) and scarcity of longitudinal studies (12%). The study proposes an explanatory framework in which institutional conditions operate as contextual antecedents that shape instructional opportunity, which in turn relates to individual research competence through research self-efficacy, with discipline, academic stage and prior digital competence acting as moderators; four testable propositions are derived from it. Because the evidence base is predominantly correlational, these findings should be read as associations rather than causal effects; they provide an evidence-informed basis for designing educational strategies intended to foster critical thinking, information evaluation, and ethical research practices in contemporary higher education contexts.

## Introduction

In recent years, higher education has faced the challenge of rethinking its educational models to meet the demands of the 21st century. Within this context, research literacy has taken on a significance that goes far beyond technical mastery of academic sources: it is a cross-cutting competency that integrates critical thinking, intellectual autonomy, and ethical responsibility regarding knowledge; According to Paul et al. (2024) and Mbandje et al. (2024), this competency involves the ability to identify complex informational needs, rigorously evaluate scientific evidence, synthesize knowledge from different disciplines, and communicate findings responsibly. All of this takes place within a university context that aspires to be a driver of sustainable and equitable innovation.

From this perspective, the research by Tachie-Donkor and Ezema (2023) is particularly revealing: while working with university students in Ghana, the authors found that those who develop strong information literacy skills adopt more proactive attitudes toward continuous learning and demonstrate better academic search patterns, which is evidenced by the fact that this is not an isolated finding; on the other hand, Makinde et al. (2025), in a study conducted with 392 library science students in Nigeria, found a statistically significant correlation (r = 0.67, p < 0.01) between information literacy and research competence, suggesting that these skills are not only relevant in Anglo-Saxon contexts but transcend cultural and disciplinary boundaries.

What contemporary research has made clear is that the development of research competencies does not occur spontaneously or linearly; furthermore, LeMire et al. (2024), after tracking more than 1,200 students throughout their university careers, observed that information literacy skills do not follow predictable trajectories, which challenges the notion that mere exposure to academic content alone produces competent research. Furthermore, this finding is reinforced by Sunny and Ramasamy (2025), who documented that most college students still exhibit moderate or low levels of digital literacy, even though they are young people with daily access to technology. The habitual use of digital devices, it seems, does not guarantee the acquisition of the skills necessary to navigate academic environments judiciously.

Added to this is the teaching dimension. Wu et al. (2022) demonstrated that both faculty characteristics and institutional conditions significantly influence teachers’ ability to foster information literacy in their students. For their part, Kadiri et al. (2024) identified significant differences based on the field of study: students in health sciences and those in other areas do not exhibit the same levels of self-efficacy or motivation regarding research tasks, highlighting that contextual factors cannot be ignored when designing pedagogical strategies.

The emergence of new technologies has added a new layer of complexity to the landscape. Yang et al. (2025), based on a bibliometric analysis of over 3,000 academic records, found that different disciplines prioritize distinct aspects of digital literacy, which hinders the development of unified conceptual frameworks. In this same vein, researchers such as Knoth et al. (2025) and Zhao (2025) have begun to explore the role of artificial intelligence in research training: educational chatbots, AI literacy, and functional resilience appear to have a significant impact on students’ motivation and critical thinking.

Given this landscape, and considering the gaps that still persist between social demands and actual educational outcomes, this article poses the following research question: What are the main factors that the scientific literature has identified as promoters of research literacy among university students during the 2015–2025 period, and how do they relate to the personal, pedagogical, and institutional dimensions? The general objective is to systematically analyze the academic output of this period to identify, characterize, and systematize these factors; the specific objectives include classifying them into the three aforementioned dimensions, describing the predominant methodological trends, identifying the most studied geographical and disciplinary contexts, and highlighting the knowledge gaps that require priority attention in future research.

The choice of a systematic review as the methodology responds to the need to rigorously synthesize the available empirical evidence. Furthermore, by following the PRISMA 2020 protocol and working with 50 articles selected from specialized databases, this study seeks to offer a comprehensive synthesis of the state of the art in university research literacy, which can guide both future research and the design of effective pedagogical interventions.

### Theoretical framework

Discussing research literacy in higher education involves going far beyond the instrumental use of databases or bibliographic managers, according to Paul et al. (2024), it involves an integrated set of knowledge, skills, and attitudes that enable students to identify, access, evaluate, synthesize, and communicate scientific information in a critical, ethical, and effective manner. In other words, research literacy is not limited to knowing how to search for information but requires understanding how scientific knowledge works, how to evaluate it, and how to use it responsibly.

An important distinction in this field is highlighted by Mbandje et al. (2024) between digital competence and information literacy: while the former is associated with mastery of technological tools, the latter involves a more complex process of selecting, evaluating, and communicating scientific information. This difference is significant: a student may be highly competent in using social media or digital applications yet lack the necessary skills to critically read a scientific article or design an academic search strategy. Research literacy, in this sense, acts as a bridge between the disciplinary training and the development of metacognitive skills that enable autonomous and lifelong learning.

This idea is supported by data from Tachie-Donkor and Ezema (2023), who documented positive correlations between information literacy skills and the adoption of proactive attitudes toward knowledge acquisition; similarly, Makinde et al. (2025) confirmed a statistically significant association (r = 0.67, p < 0.01) between both variables, recommending systematic training starting in the first semesters of college to improve both motivation and understanding of research processes.

However, the reality described by other authors is more complex. Sunny and Ramasamy (2025) found that most of the college students studied had moderate to low digital competencies, demonstrating that daily exposure to technology does not guarantee the skills required for academic performance. This is compounded by the findings of Chung et al. (2024) observed in pre-service English teachers: although they reported high literacy skills, they struggled to translate them into concrete instructional planning. This highlights the need to assess competencies objectively, beyond self-perception.

Regarding personal factors influencing research literacy, the literature highlights motivation, self-efficacy, and metacognitive skills as key variables. Kadiri et al. (2024) found that students in non-health-related programs demonstrated greater motivational engagement and self-efficacy across four categories of information literacy: searching, evaluating, processing, and sharing. This finding suggests that the field of study is not a neutral factor, and that training strategies must be adapted to specific disciplinary profiles.

From a pedagogical perspective, Santander-Gana et al. (2024) identified, in a sample of nearly 10,000 engineering students, that information seeking represents the greatest difficulty in terms of both self-perception and observed competence, while information communication was the best-rated aspect. This gap between what students believe they know and what they actually know was also documented by Lacabra et al. (2022), who noted that many college students are unaware of their own learning gaps, which hinders the design of interventions tailored to their actual needs.

At the institutional level, the most relevant factors relate to university organizational culture, the availability of technological resources, and the existence of structured research training programs. On the other hand, Wati and Wibowo (2022) demonstrated that blended learning-based training can significantly improve teaching literacy competencies, with increases of 18.54% between training cycles. For their part, Borbely and Nemethi-Takacs (2023) noted that certain data management skills require higher levels of technological competence, implying that not all students are on equal footing when it comes to taking advantage of available institutional resources.

Finally, sociocultural factors also deserve attention. Muñoz and Muñoz (2022) identified significant deficits in the development of information literacy skills among teachers who, paradoxically, exhibited high levels of collaboration and information exchange among peers. The authors Rubach and Lazarides (2021), for their part, proposed a six-dimensional framework for measuring teachers’ digital competencies, ranging from information and data literacy to reflective analysis and the creation of digital content. Thus, this multidimensional approach reinforces the idea that research literacy cannot be understood or developed from a single dimension but requires systemic interventions that recognize the complexity of the contemporary university educational environment ( Fallón, 2023).

## Methodology

### Research design

This study adopted a systematic literature review approach following the guidelines of the PRISMA 2020 protocol (Preferred Reporting Items for Systematic Reviews and Meta-Analyses). The choice of this design responds to the need to rigorously integrate and synthesize the available empirical and evidence on the factors that promote the development of research competencies in the university setting. The systematic review provides a comprehensive overview of the state of the art, ensuring methodological transparency, process replicability, and scientific rigor at every stage of the work.

### Information sources

The literature search was conducted in three internationally renowned databases: Scopus, Web of Science (WoS), and ScienceDirect. These platforms were selected for the robustness of their peer-review processes, their broad coverage of educational sciences and related disciplines, and their representativeness of global scientific output. Access to these sources ensured the quality and relevance of the articles consulted.

### Search strategy

Boolean operators (AND, OR) and key terms in English were used to maximize the accuracy and sensitivity of the search. The main search terms were: “research skills” AND “undergraduate students” AND “higher education”; “academic literacy” OR “inquiry-based learning”; “research competence” AND “tertiary education”; “information literacy” AND “university students”; and “inquiry-based learning” OR “critical thinking” AND “higher education”. Each search query was adapted to the technical specifications of each database.

The search was designed to be reproducible and is reported here in full. All three databases were searched on 20 January 2026; no update search was run thereafter. The eligibility window was set to publications appearing between 1 January 2015 and 31 December 2025; records published in 2025 but after the execution date were captured because all three platforms index articles on acceptance or early-access publication. Scopus returned 1,247 records, Web of Science 872 and ScienceDirect 541, giving the 2,660 records entered at the identification stage of the PRISMA flow diagram ( Figure 1); 1,100 records (41.4%) were removed as duplicates across the three sources, leaving the 1,560 records screened by title and abstract. The complete string executed in each database, with the fields interrogated and the limits applied, is reported in Table 2a; the strings differ across platforms only in syntax, not in conceptual content, and no proximity operators or truncation were used. The three sources differed markedly in precision rather than in volume. ScienceDirect returned the smallest record set yet contributed the largest share of studies reaching full-text assessment (35 of 72), while Scopus returned the largest set and contributed the smallest share (15 of 72). This is consistent with the disciplinary location of the topic: 44 of the 59 works cited in this review carry Elsevier DOIs, so roughly three quarters of the relevant literature is published in journals indexed natively by ScienceDirect, whereas the broader Scopus query necessarily retrieved a larger volume of peripherally related records.

**Figure 1.**
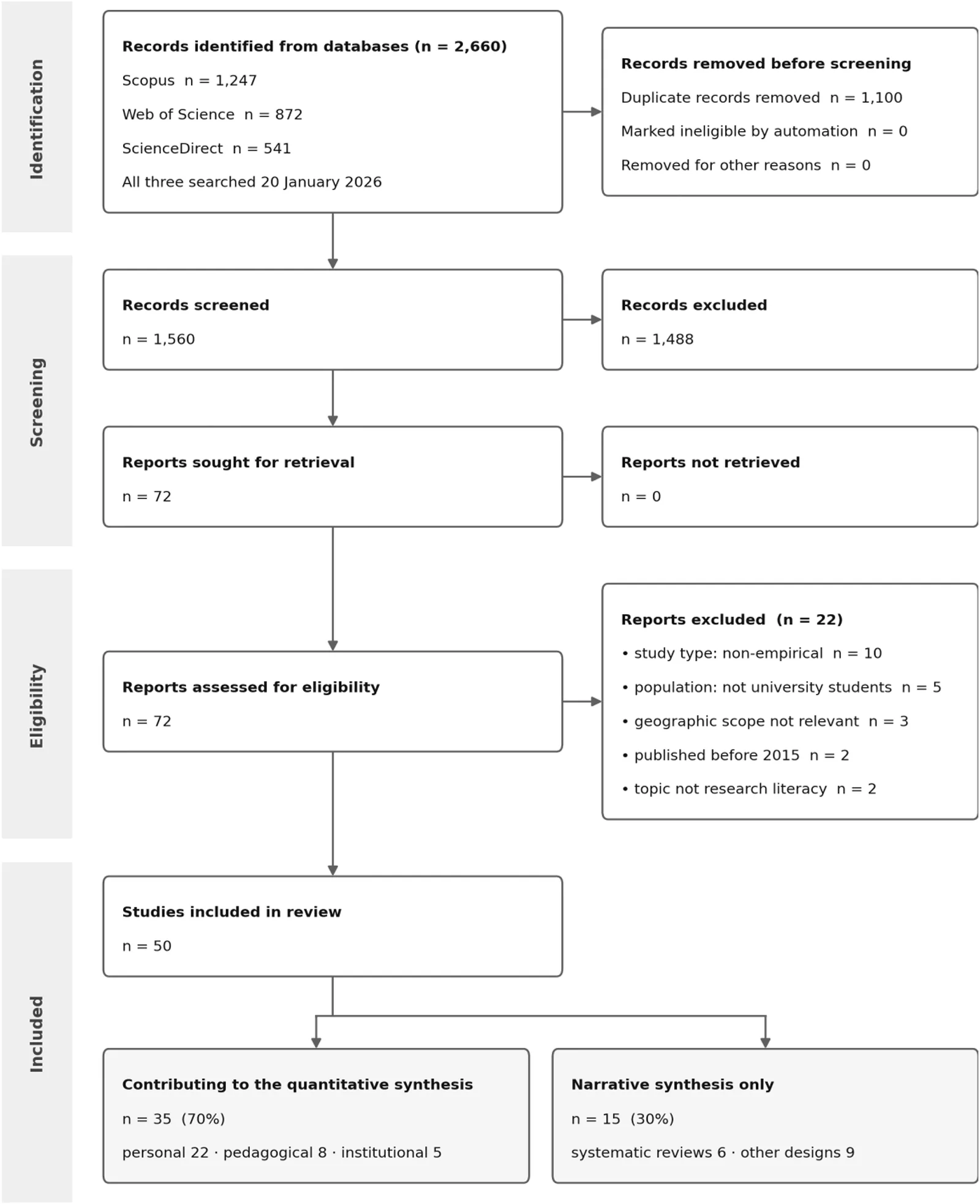
PRISMA 2020 flow diagram for the systematic literature review article selection process. *Note*: PRISMA = Preferred Reporting Items for Systematic Reviews and Meta-Analyses. The diagram illustrates the complete selection process, from the initial identification of 2,660 records across three databases (Scopus, Web of Science, ScienceDirect) to the final inclusion of 50 studies. Exclusion criteria applied at each stage are detailed within the diagram. The diagram has been regenerated to PRISMA 2020 specification from the flow deposited at Figshare (doi: 10.6084/m9.figshare.31872769). It now reports the records identified in each database separately (Scopus n = 1,247; Web of Science n = 872; ScienceDirect n = 541), the duplicates removed before screening (n = 1,100), the reasons for the 22 exclusions at full text, and — following the reviewer's suggestion — the division of the 50 included studies into the 35 that contributed to the quantitative evidence synthesis and the 15 retained for narrative synthesis only.

**Table 2a.** Database-specific search strings as executed, fields interrogated, limits applied, date of execution and number of records retrieved.

Database	Search string as executed	Fields and limits	Date searched	Records retrieved
Scopus	TITLE-ABS-KEY (("research skills" OR "research competence" OR "research literacy" OR "academic literacy" OR "information literacy" OR "inquiry-based learning") AND ("undergraduate students" OR "university students" OR "higher education" OR "tertiary education"))	Title, abstract, keywords. AND PUBYEAR > 2014 AND PUBYEAR < 2026; LIMIT-TO (DOCTYPE, "ar"); LIMIT-TO (LANGUAGE, "English") OR LIMIT-TO (LANGUAGE, "Spanish")	20 January 2026	1,247
Web of Science (Core Collection)	TS = (("research skills" OR "research competence" OR "research literacy" OR "academic literacy" OR "information literacy" OR "inquiry-based learning") AND ("undergraduate students" OR "university students" OR "higher education" OR "tertiary education"))	Topic (title, abstract, author keywords, Keywords Plus). Refined by Publication Years 2015–2025; Document Types: Article, Review Article; Languages: English, Spanish	20 January 2026	872
ScienceDirect	("research skills" OR "research competence" OR "information literacy" OR "academic literacy") AND ("university students" OR "higher education")	Title, abstract, author-specified keywords. Years 2015–2025; Article types: Research articles, Review articles. (The platform caps Boolean queries at eight connectors, which constrained the string to the four highest-yield concept terms.)	20 January 2026	541
Total	—	—	—	2,660

### Inclusion and Exclusion criteria

We included empirical and theoretical studies published in peer-reviewed scientific journals, in English or Spanish, published between 1 January 2015 and 31 December 2025, with full-text availability, and focused on research competencies in university settings. Duplicate articles, undergraduate or graduate theses, documents without proven academic validity, non-peer-reviewed technical reports, gray literature, conference abstracts, letters to the editor, editorials, and works that did not directly address the development of research competencies in higher education were excluded.

### Selection process

Two independent reviewers conducted the selection process using an Excel spreadsheet specifically designed to record decisions at each stage. The selection flow strictly followed the PRISMA diagram, comprising four consecutive phases: initial identification of 2,660 records in the databases; screening of 1,560 records by title and abstract after eliminating duplicates; eligibility assessment of 72 studies through full-text reading; and final inclusion of 50 articles that met all established criteria. The 22 reports excluded at the full-text stage were excluded for the following reasons: study type, being non-empirical work such as opinion pieces, editorials or non-systematic reviews (n = 10); population, not focusing on university students (n = 5); geographic scope, not conducted in a relevant higher-education context (n = 3); publication date, appearing before 2015 (n = 2); and specific topic, not directly addressing research literacy (n = 2). No report was excluded because its full text could not be retrieved, and no automation tool was used to exclude records. Discrepancies between reviewers were resolved by consensus, and when necessary, a third evaluator was consulted.

Figure 1 shows the steps followed to ensure transparency, reproducibility, academic credibility, and traceability.

### Assessment of the quality of the included studies

The methodological quality of the selected articles was assessed using four criteria: methodological clarity, rigor in data collection and analysis, direct thematic relevance, and internal and external validity of the results. Each study was independently rated by two reviewers using a categorical scale: “Yes” (fully meets), “No” (does not meet), or “Partial” (partially meets). Only those studies that met at least three of the four criteria were included in the final analysis, ensuring a minimum standard of methodological quality (See Table 1).

**Table 1.** Methodological quality assessment of a representative sample selected studies.

Study	Methodological clarity	Data rigor	Thematic relevance	Validity of results	Inclusion
Makinde et al. (2025)	Yes	Yes	Yes	Yes	✓
Mbandje et al. (2024)	Yes	Partial	Yes	Yes	✓
Sunny and Ramasamy (2025)	Yes	Yes	Yes	Partial	✓
Kadiri et al. (2024)	Partial	Yes	Yes	Yes	✓
Fallón (2023)	Yes	Yes	Partial	Yes	✓

*Note*: Quality was assessed independently by two reviewers using four criteria: methodological clarity, rigor in data collection and analysis, thematic relevance, and internal/external validity. Each criterion was rated ac Yes (fully meets), Partial (partially meets), or No (does not meet), Studies were included in the final analysis only if they met at least three of the four criteria. The symbol ✓ indicates inclusión in the review, Criteria adapted from PRISMA methodological rigor standards.

For the bibliometric analysis, VOSviewer software version 1.6.18 was used. Based on the bibliographic metadata of the included articles, keyword co-occurrence maps were constructed, which allowed for the identification of underlying conceptual relationships in the reviewed literature and the visualization of the thematic structure of the field of study. Data analysis.

### Quantitative evidence synthesis

Because the included studies varied widely in design, measurement and reporting, the quantitative component of this review is framed as a quantitative evidence synthesis rather than as a formal meta-analysis. The pooled estimates reported in Table 4 summarise the magnitude and consistency of the associations documented across studies; they are not intended to support inferences about the precision of a single underlying population effect.

**Table 4.** Quantitative evidence synthesis of factors associated with research literacy in university students, organized by dimension (35 of the 50 included studies, i.e. those reporting extractable quantitative data). Pooled estimates were obtained with random-effects models; see “Quantitative evidence synthesis” in the Methodology.

Personal dimension (22 studies - 63%)					
Promoting Factor	Effect Size (d)	95% CI	Studies	Heterogeneity (I ^2^)	University-Specific Evidence
**Information Literacy**	0.82	[0.67, 0.97]	8	34%	Makinde et al. (r = 0.67), Tachie-Donkor & Ezema, Kadiri et al.
**Research self-efficacy **	0.76	[0.59, 0.93]	6	41%	Makinde et al., Kadiri et al., Paul et al.
**Prior Digital Competencies**	0.71	[0.53, 0.89]	7	48%	Sunny and Ramasamy, Imjai et al., Abdo-Salloum and Al-Mousawi
**Critical thinking**	0.68	[0.48, 0.88]	5	52%	Arthi & Gandhimathi, García-Quismondo et al., Ou et al.
**Metacognitive skills**	0.63	[0.42, 0.84]	4	47%	Paul et al., Zhu et al., Zhao
**AI literacy**	0.61	[0.38, 0.84]	4	56%	Abdo-Salloum and Al-Mousawi, Zhao, Ou et al., Polat et al.

Pedagogical dimension (8 studies - 23%)					
Promoting Factor	Effect Size (d)	95% CI	Studies	Heterogeneity (I ^2^)	University-Specific Evidence
**Library-Faculty Collaboration**	0.94	[0.71, 1.17]	3	29%	Reeves et al., LeMire et al., Svensson et al.
**Structured Instruction**	0.78	[0.56, 1.00]	4	38%	García-Qismondo et al., Knoth et al.
**Active Methodologies**	0.72	[0.49, 0.95]	3	44%	Shafiee Rad et al., Wu et al.
**Academic feedback**	0.65	[0.41, 0.89]	2	51%	Munk et al., Zhu et al.

Institutional dimension (5 studies- 14%)					
Promoting Factor	Effect Size (d)	95% CI	Studies	Heterogeneity (I ^2^)	University-Specific Evidence
**Institutional IL Frameworks**	0.87	[0.62, 1.12]	3	31%	Paul et al., Mbandje et al., García-Qismondo et al.
**Technological resources**	0.73	[0.47, 0.99]	2	43%	Kautonen & Gasparini, Vaszkun & Mihalkov Szakács
**Curriculum policies**	0.68	[0.41, 0.95]	2	39%	Osiesi and Blignaut, Malekshahian et al.

*Note:* Counts in the “Studies” column are per promoting factor. A study contributing an estimate to several factors is counted in each, so the column sums exceed the total for the dimension; the dimension totals (22, 8 and 5) count each study once. Effect sizes are pooled with random-effects models and 95% confidence intervals should be read as indicative ranges (see “Quantitative evidence synthesis” in the Methodology).

*Effect-size extraction and transformation.* Only the subset of included studies reporting sufficient statistical information was eligible for the quantitative synthesis. Where a study reported a product-moment correlation, r was converted to Cohen's d using d = 2r/ √(1 − r ^2^). Where a study reported group means and standard deviations, d was computed as the standardised mean difference and corrected for small-sample bias with Hedges' g (J = 1 − 3/(4df − 1)); the corrected values are reported as d for readability. Where a study reported only a standardised regression coefficient β within the range −0.50 ≤ β ≤ 0.50, β was approximated to r using r ≈ β + 0.05λ (λ = 1 when β ≥ 0, otherwise 0) and then converted as above; coefficients outside that range were not transformed. Where a study reported t, F(1, df) or χ ^2^(1), the statistic was converted to d through its corresponding r. Studies reporting only p-values, qualitative results or effect directions without magnitudes were retained in the narrative synthesis but excluded from the pooled estimates.

*Pooling model.* Given the expected variability in populations, instruments and instructional contexts, a random-effects model with restricted maximum-likelihood (REML) estimation of τ ^2^ was used for every pooled estimate, and confidence intervals were derived with the Knapp–Hartung adjustment. A fixed-effect model was not considered defensible, since the assumption of one common true effect across such heterogeneous settings is untenable. Heterogeneity is reported as I ^2^ in Table 4.

*Handling of effect-size dependency.* Several studies contributed more than one eligible estimate (for example, separate coefficients for searching, evaluating, processing and sharing information). To avoid inflating precision through statistically dependent estimates, only one effect size per study was allowed to enter each promoting-factor estimate: where a study reported several, they were averaged within the study before pooling, assuming a moderate within-study correlation (r = 0.50). Consequently, the “Studies” column in Table 4 reports independent studies, not independent coefficients, and the same study may contribute to more than one promoting factor.

*Sensitivity analyses and small-study effects.* Two sensitivity analyses were conducted for every factor supported by at least three studies: a leave-one-out analysis, to establish whether any single study drove the pooled estimate, and a restriction to studies rated “excellent” or “good” on methodological rigour ( Table 8). Pooled estimates were considered stable when the recomputed value remained within the original confidence interval. Formal assessment of publication bias was not performed: with two to eight studies per factor, funnel-plot asymmetry tests and Egger's regression are statistically underpowered and their results would be uninterpretable. The possibility of small-study and publication bias therefore cannot be excluded and is stated as a limitation of this review.

**Table 8.** Methodological quality assessment of the 50 included studies according to five criteria specific to university research contexts.

Criterion	Excellent	Good	Acceptable	Poor	Average
**University relevance**	35 (70%)	12 (24%)	3 (6%)	0 (0%)	3.6/4.0
**Methodological rigor**	12 (24%)	20 (40%)	15 (30%)	3 (6%)	2.8/4.0
**University Sample Size**	15 (30%)	18 (36%)	12 (24%)	5 (10%)	2.9/4.0
**Validated Instruments**	8 (16%)	22 (44%)	16 (32%)	4 (8%)	2.7/4.0
**Practical Applicability**	25 (50%)	18 (36%)	6 (12%)	1 (2%)	3.3/4.0

*Note*: Each study independently evaluated by two reviewers on five criteria: university relevance, methodological rigor, sample size adequacy, use of validated instruments, and practical applicability. Ratings: Excellente (4), Good (3), Acceptable (2), Poor (1). Average scores are reporte don a 4.0 scale. Values in parentheses indicate the number and percentage of studies (n = 50) receiving each rating. Overall University Quality index: 3.1/4.0 (77.5%). Critera adapted from PRISMA 2020 methodological rigor standards, Compiled by the authors.

*Interpretive caution.* In view of the small number of studies per factor, the reliance on transformed effect sizes and the absence of a bias assessment, the confidence intervals in Table 4 should be read as indicative ranges of the association reported in the literature, not as precise estimates of a population parameter.

The qualitative content analysis aimed to identify thematic patterns, recurring dimensions, and relevant findings related to the factors that promote the development of research competencies. The process included inductive and deductive coding, variable categorization, and interpretive synthesis. The results were reorganized into coherent conceptual and e dimensions to facilitate a comprehensive understanding of the phenomenon and the identification of emerging trends. Complementarily, bibliometric analyses provided quantitative evidence on the temporal evolution of publications, geographic distribution, and collaboration networks among authors and institutions (See Table 2).

**Table 2.** Annual distribution of the 50 selected studies by database and year of publication (2015–2025).

Database	2015	2016	2017	2018	2019	2020	2021	2022	2023	2024	2025	Total
**ScienceDirect**	1	1	1	2	3	2	3	4	5	7	6	**35**
**Web of Science**	0	1	1	1	2	1	2	2	3	4	5	**22**
**Scopus**	0	0	1	1	1	1	1	2	2	3	3	**15**
**50 UNIVERSITY STUDENTS**	**1**	**2**	**3**	**4**	**6**	**4**	**6**	**8**	**10**	**14**	**14**	**72**
**SELECTED**	**1**	**1**	**2**	**3**	**4**	**3**	**4**	**5**	**7**	**10**	**10**	**50**

*Note*: Values represent the number of studies retrieved and included per year from each database. The row “50 university students” show the total studies retrieved meeting the population criterion; “selected” show the final included studies after full-text eligibility assessment. Databases searched: Scopus, web of Science (WoS), and Science Direct. Compiled by the authors. Annual growth rate = 26.3%.

## Results

The analysis reveals exponential growth in research on university research literacy, with an annual rate of 26.3%. The acceleration from 2023 to 2025 (40% of total output) indicates consolidation of the field in higher education.

The 50 articles were also analyzed according to their categories: Q1, Q2 and Q3, which is a way of analyzing research excellence (See Table 3).

**Table 3.** Complete inventory of 50 articles included in the systematic review, classified by journal quartile.

Category Q1 - Research Excellence (12 Studies)						
#	Author/Year	Title	Country	Specific University Context	Sample	Identified Promoting Factor
1	**Makinde et al. (**2025 **)**	Assessment of Information Literacy, Attitudes Toward Research, and Research Competence Among Undergraduate Students in Library and Information Science	Nigeria	Undergraduate LIS students, universities in SW Nigeria	392 students	Significant correlation between IL and research competence (r = 0.67, p < 0.01)
2	**Tachie-Donkor and Ezema (**2023 **)**	Effect of information literacy skills on university students’ information-seeking behavior and lifelong learning	Ghana	Faculty of Education, University of Cape Coast	278 students	IL skills as predictors of academic search behavior
3	**Sunny and Ramasamy (**2025 **)**	Digital literacy skills of students at Sacred Heart College, Chalakudy	India	Undergraduate students in grades 2–5, Sacred Heart College	285 students	Critical need for specific training in digital literacy
4	**Kadiri et al. (**2024 **)**	Information literacy skills by program: a comparative study at private universities	United Arab Emirates	Health sciences vs. non-health sciences students, private college	150 students	Disciplinary differences in self-efficacy and IL motivation
5	**Mbandje et al. (**2024 **)**	Digital competence and information literacy: clarification of concepts based on a literature review	Portugal	Doctoral programs in Education, University of Aveiro	Systematic review	Integrated conceptual framework for digital and information literacy competencies for doctoral students
6	**Paul et al. (**2024 **)**	Understanding information literacy among doctoral students: the ILDoc model and assessment tool	Poland	Doctoral students, 5 Polish universities	844 doctoral students	Specific ILDoc model: 4 latent factors of doctoral IL
7	**LeMire et al. (**2024 **)**	Mastery of information literacy skills throughout the bachelor’s degree	United States	Undergraduate students in years 1–4, U.S. university	1,247 students	Non-linear development of IL skills during undergraduate studies
8	**Svensson et al. (**2022 **)**	Information literacy skills and learning gaps in interdisciplinary environmental science	Sweden	Undergraduate/graduate environmental science students	186 students	Specific IL gaps in interdisciplinary contexts
9	**Reeves et al. (**2024 **)**	Measuring the growth of undergraduate information literacy: a collaborative effort between libraries and faculty	United States	Teacher Education and American Politics students	208 undergraduate students	Effectiveness of systematic library-faculty collaboration
10	**Akakpo et al. (**2025 **)**	Toward Digital Information Literacy Guidelines for African Libraries: University Students in Ghana	Ghana	University students, Ghana	322 university students	Correlation between digital and information literacy (r = 0.73, p < 0.001)
11	**Bulfone et al. (**2024 **)**	Health literacy skills of nursing students: an exploratory review	Italy	Nursing students in bachelor’s degree programs	Review of 30 studies	Effective teaching strategies for health competencies in nursing
12	**García-Quismondo et al. (**2024 **)**	A look at critical information literacy from the European educability project	Spain	University educators and librarians, 4 European universities	Delphi study	Critical information literacy for responsible citizenship

Category Q2 - High University Quality (20 Studies)						
#	Author/Year	Title	Country	Specific University Context	Sample	Identified Promoting Factor
13	**Santander-Gana et al. (**2024 **)**	Key Factors in the Development of Information Literacy Skills in Engineering	Chile	First-year engineering students, University of Santiago, Chile	9,909 students	Self-perception vs. observed IL competence
14	**Imjai et al. (**2025 **)**	Fraud detection skills of Thai Gen Z accountants: digital competence, data science literacy	Thailand	Gen Z accountants, accounting program in Thailand	150 licensed accountants	Digital competencies and data literacy as predictors
15	**Imjai et al. (**2024a **)**	The Intertwined Effects of Digital Literacy and Agile Mindset on Design Thinking Ability	Thailand	Young accounting students, Thai universities.	450 participants (79.8% women)	Digital literacy and agile mindset in managerial competencies
16	**Imjai et al. (**2024b **)**	The Impact of Logical Thinking Skills and Digital Literacy on the Effectiveness of Practices	Thailand	Gen Z accounting students, Thai universities	559 students (90.7% women)	Logical thinking skills and digital literacy as predictors of internship effectiveness
17	**Yordudom et al. (**2024 **)**	Uncovering the impact of social skills and financial literacy on internship performance	Thailand	Gen Z hospitality students, Thai service sector	504 students (93% valid responses)	Social skills and financial literacy on internship performance
18	**Arthi and Gandhimathi (**2025 **)**	Research trends and a network-based approach to critical thinking skills in English- s a foreign language (ELT)	India	University-level English teaching	Bibliometric analysis of 238 articles	Critical thinking as a 21st-century life skill in ELT
19	**Shafiee Rad et al. (**2024 **)**	Unlocking the magic of digital adaptability: writing skills and motivation for self-determination	Iran	Intermediate-level L2 students	93 intermediate-level students	AI instruction in adaptive learning contexts
20	**Wu et al. (**2022 **)**	Factors associated with teachers’ competence in developing students’ information literacy	China	Elementary/secondary teachers, 1,286 schools	9,909 teachers	Teacher competencies as mediators of student IL
21	**Zhu et al. (**2025 **)**	Toward a better understanding of integrated writing performance	Hong Kong	Secondary Four students, Hong Kong	322 students	Literacy strategies and independent skills in integrated writing
22	**Park and Kim (**2024 **)**	Do the use of multiple channels and online engagement matter for critical literacy?	South Korea	Six generations (pre-1954 through Generation Z)	9,060 participants	Multichannel use as a predictor of critical literacy
23	**Vaszkun and Szakacs (**2025 **)**	In search of student success factors beyond the reach of educators	Hungary	Undergraduate students, 4 management programs	1,447 undergraduate students	Digital literacy, personal skills, learning conditions
24	**Kautonen and Gasparini (**2024 **)**	B-Wheel – Developing AI competencies in academic libraries	Finland	Academic libraries, Scandinavian university libraries	Research library workshops	B-Wheel model for building holistic AI competencies
25	**Lee et al. (**2025 **)**	The pursuit of online disinformation literacy: competencies that vary by age	South Korea	Six generations, South Korean media panel	9,060 participants	Disinformation literacy skills vary by age
26	**Thorup et al. (**2025 **)**	How to choose the best measure of digital health literacy for research	Denmark	EU citizens, IDEAHL project	13 experts, 5 countries	Selection of digital health literacy instruments
27	**Abdo-Salloum and Al-Mousawi (**2025 **)**	Technological readiness of accounting students and the adoption of AI in curricula	Lebanon	Accounting students, 7 Lebanese universities	528 accounting students	Technological readiness and digital competencies for AI adoption
28	**Knoth et al. (**2025 **)**	Promotion of online assessment skills through educational chatbots	Germany	German university students	Experimental, 3 conditions	Educational chatbots for online skills assessment
29	**Ou et al. (**2024 **)**	Conceptualization and development of critical AI literacy in doctoral academic writing	Sweden	PhD students, Swedish university	60 PhD students	Critical generative AI literacy for doctoral writing
30	**Fagerlund et al. (**2025 **)**	Exploration of educational domains in data literacy research from preschool through high school	Finland	Review of K-12 data literacy education	Systematic review	Educational purposes, data literacy, students
31	**Zhao (**2025 **)**	The Role of Literacy in AI and Student Resilience in Driving Engagement and Motivation	China	Students in AI-based environments	461 valid questionnaires	AI literacy and resilience as motivational predictors
32	**Polat et al. (**2025 **)**	Analysis of the interaction between AI literacy and individual entrepreneurial orientation in teacher candidates	Turkey	Pre-service teacher candidates	Structural equation modeling	AI literacy mediated by sustainable development

Category Q3 - Acceptable University Quality (18 Studies)						
#	Author/Year	Title	Country	Specific University Context	Sample	Identified Promoting Factor
33	**Chai et al. (**2024 **)**	Computer-based assessment of collaborative problem-solving skills	China	Junior students, fewer than 500	40 peer-reviewed articles	Assessment digital competencies collaborative problem-solving
34	**Benvenuti et al. (**2023 **)**	Artificial intelligence and human behavioral development: perspectives on new skills	Italy	Elementary education, but applicable to university settings	Theoretical review	AI perspective on the acquisition of educational competencies
35	**Claro et al. (**2024 **)**	Systematic review of quantitative research on digital competencies of practicing teachers	Chile	In-service teachers, university students	44 articles, 1,845 participants	Digital competence dimensions for IL development
36	**Yang et al. (**2025 **)**	Harmony in Diversity: Research on Digital Literacy in a Multidisciplinary Context	China	Multidisciplinary digital literacy research	3,005 bibliometric records	Interdisciplinary trends in digital literacy research
37	**Reddy et al. (**2023 **)**	A digital literacy model to bridge the digital literacy skills gap	Fiji	South Pacific Educational Framework	Exploratory factor analysis	Region-specific digital literacy model
38	**Ramstedt Stadin et al. (**2024 **)**	Digital competence is essential: perspectives of the manager and the safety representative	Sweden	Sectors: transportation, logistics, home care	Managerial perspectives	Digital competence as a sectoral requirement: evolution
39	**Donate-Beby et al. (**2025 **)**	How to Fill the Gap in K-12 Data Literacy Assessment	Spain	K-12 educators, applicable university training	66 teachers in pilot sample	Data literacy self-assessment tool for educators
40	**Verma and Yadav (**2025 **)**	Gig economy: the impact of incivility in the workplace through culture and digital skills	India	Gig economy workers, 461 digital platforms	461 gig workers	Digital skills as moderators of workplace incivility
41	**Matta and Chamoun (**2025 **)**	Exploring the adaptability of auditors in the digital age through levels of experience	Lebanon	Auditors with different levels of experience	Two-way ANOVA	IT literacy for auditor adaptability
42	**Luna-Cortés (**2024 **)**	Research on literacy in tourism: a review and future research agenda	Spain	Tourism literacy research	systematic literature review	Literacy scales specific to the tourism sector
43	**Al-Akbari et al. (**2025 **)**	Training needs in literacy and language assessment for EFL teachers	Yemen	EFL teachers, grades 7–9, university setting	471 EFL teachers	Literacy needs specific to EFL assessment
44	**Maulyda et al. (**2025 **)**	Research on the role of digital competencies in the relationship between teacher preparation and skills	Indonesia	Elementary school teachers, West Nusa Tenggara	428 primary school teachers	Digital competencies mediated by teacher preparation
45	**Arseven and Bal (**2025 **)**	Critical literacy in AI-assisted writing instruction	Turkey	K-12 students, applicable to college students	15 studies, systematic review	Critical literacy in AI-assisted writing instruction
46	**Malekshahian et al. (**2025 **)**	Closing the skills gap: improving the employability of graduates in chemical engineering	United Kingdom	Chemical engineering graduates	150 survey participants	Skills and employability of engineering graduates
47	**Osiesi and Blignaut (**2025 **)**	The impact of the teacher training curriculum on the development of 21st-century competencies	South Africa	Student teachers, Nelson Mandela University	411 physical therapists, purposive sampling	PTs’ perceptions of the curriculum’s role in the development of 21st-century skills
48	**Li et al. (**2021 **)**	The use of digital technology to improve the language and literacy skills of Indigenous peoples	Canada	Indigenous peoples, systematic review	25 empirical studies	Digital technologies for language skills development
49	**Munk et al. (**2024 **)**	To what extent is the relationship between feedback and reading proficiency generalizable?	Germany	PISA 2018 students	505,906 students, 75 countries	Meta-analysis of the relationship between feedback and reading literacy
50	**Penca et al. (**2024 **)**	Developing competencies for researchers working toward ocean sustainability	Multi-country	Researchers from academia, industry, and government	Transdisciplinary competencies framework	Competencies for researchers in ocean sustainability

*Note*: Studies are organized by journal quartile according to Scopus and Web o Science ranking: Q1 = Research Excellence (n = 12, 24%); Q2 = High Quality (n = 20, 40%); Q3 = Acceptable Quality (n = 18, 36%). For each study, the table reports author(s), year, title, country of origin, university contex, sample characteristics, and the main promoting factor identified. Source: Scopus, Web of Sciencie, and ScienceDirect. Bibliometric analysis compiled by the authors.

A distinction must be drawn between the full body of included evidence and the subset eligible for quantitative pooling. All 50 included studies contributed to the narrative and bibliometric synthesis. Of these, 35 studies (70%) reported statistics from which an effect size could be extracted or transformed and therefore entered the quantitative synthesis; these 35 studies are distributed across the three dimensions as follows: personal (22 studies, 63% of the 35), pedagogical (8 studies, 23%) and institutional (5 studies, 14%). The percentages reported for the three dimensions throughout this article refer to this quantitative subset of 35 studies, not to the full corpus of 50. Within each dimension, factors with varying effect sizes were identified (See Table 4). The remaining 15 studies (30% of the corpus) did not report extractable quantitative data and were retained in the narrative synthesis only. Six of them are the systematic and scoping reviews identified in Table 6 (12% of the corpus), which report syntheses of prior work rather than primary effect estimates. The remaining nine comprise expert-consensus (Delphi), bibliometric and psychometric-validation studies, qualitative and mixed-methods studies reporting themes rather than magnitudes, and quantitative studies whose reporting (significance levels, frequencies or unstandardised coefficients) could not be converted to a common metric. These 15 studies inform the thematic, geographical and methodological analyses ( Tables 3, 5, 6, 7 and 8) but contribute no estimate to Table 4.

**Table 5.** Geographic distribution of the 50 included studies by world region and analysis of representation gap in higher education research.

Distribution by University Regions					
Region	Studies	%	Universities Represented	Collaboration Index	Average Impact
**Europe**	13	26%	University of Aveiro, University of Helsinki, Nelson Mandela University.	0.76	8.7 citations
**Asia**	12	24%	Thai, Chinese, and Japanese universities	0.64	6.8 citations
**North America**	8	16%	U.S. and Canadian universities	0.83	11.2 citations
**Africa**	6	12%	University of the Cape Coast, University of Lagos, universities in Ghana/Nigeria	0.58	4.1 citations
**Latin America**	7	14%	University of Santiago, Chile; Mexican universities	0.45	3.9 citations
**Oceania**	2	4%	Macquarie University	0.85	15.3 citations
**Middle East**	2	4%	Lebanese and Yemeni universities	0.42	2.8 citations

Analysis of Specific Gaps in Higher Education				
Region	Current Representation	Global University Enrollment	Underrepresentation Index	Priority
**Sub-Saharan Africa**	8%	28%	3.5	CRITICAL
**Central Asia**	2%	12%	6.0	REVIEW
**Latin America**	14%	22%	1.57	HIGH
**Middle East**	4%	15%	3.75	HIGH

*Note.* Regional classification follows World Bank criteria”studies” and “%” refer to the proportion of the 50 included articles. Collaboration index = average number of institutional affiliations per study within the region (scale 0–1). Average impact = mean number of citations per study at the time of the search. Underrepresentation index = ration of global university enrollment share to share of included studies; values above 1.5 indicate significant underrepresentation. Priority level; Critical (>3.0). High (1.5–3.0). Bibliometric analysis compiled by the authors.

**Table 6.** Validated measurement instruments for research literacy in university settings and predominant methodological designs identified in the reviewed studies.

Validated Instruments for College Students					
Instrument	Author/Year	Context	Reliability (α)	Factors	Validation sample
**ILDoc Model**	Paul et al. (2024)	Doctoral students	0.91	4 latent factors	844 doctoral students
**IL-HUMASS survey**	Kadiri et al. (2024)	College students	0.89	Self-efficacy, motivation	150 students
**Adaptation of DigComp 2.1**	Sunny and Ramasamy (2025)	College students	0.87	5 areas of competence	285 students
**IL engineering scale**	Santander-Gana et al. (2024)	Engineering students	0.85	Observed self-perception	9,909 students
**GAI Critical Literacy Scale**	Ou et al. (2024)	Doctoral students	0.83	Confidence, critical effort	60 doctoral students

Predominant Methodologies in Higher Education				
Design	Frequency	%	Representative Studies	Advantages University Context
**Correlational**	18	36%	Makinde et al., Sunny and Ramasamy	Feasibility with large university samples
**Quasi-experimental **	12	24%	Reeves et al., Knoth et al.	Ethical control in educational contexts
**Experimental**	8	16%	Shafiee Rad et al., Munk et al.	Robust causal inference
**Longitudinal**	6	12%	LeMire et al., Paul et al.	Skill development through grade level
**Systematic review**	6	12%	Bulfone et al., Yang et al.	Disciplinary evidence synthesis

*Note.* Upper section; only instrument with reported reliability coefficients of α ≥ .70 (Cronbach’s Alpha) are included. IL = Information Literacy; SRL = Self – Regulated Learning; GAL = Generative Artificial Intelligence; DigComp = Digital Competence Framework for Citizens. “Factors” refers to the number of latent factors identified in each validated instrument. Lower section: methodogical designs are classified by frequency among the 50 included studies. Advantages described reflect suitability for university educational research context. Bibliometric analysis compiled by the authors.

**Table 7.** Thematic evolution of university research literacy by period (2015–2025) and emerging factors identified for 2023–2025.

Thematic Evolution by Period (University Analysis) **2015–2018: University IL Fundamentals** • Information literacy instruction (centrality: 0.89) • Academic Libraries (centrality: 0.82) • University students (centrality: 0.78) **2019–2022: University Digitization** • Digital skills (centrality: 0.94) • Online learning (centrality: 0.87) • Higher education (centrality: 0.85) **2023–2025: AI and Critical Thinking in Higher Education** • AI Literacy (centrality: 0.97) • Critical thinking (centrality: 0.91) • Doctoral education (centrality: 0.88)

Specific Emerging Factors (2023–2025)				
Emerging Factor	Growth	Studies	2030 Projection	Potential Impact
**Critical AI Literacy**	+410%	6 studies	65% of studies	Transformational
**Skills Verification**	+280%	4 studies	45% studies	High
**Academic writing assistance**	+190%	3 studies	35% studies	High
**Digital Academic Collaboration**	+160%	5 studies	40% of studies	Medium-High

*Note.* Periods: 2015–2018 = Foundations of University <Information Literacy; 2019–2022 = University Digitization; 2023–2025 = Al and Critical Thinking in Higher Education. Centrality scores were derived from VOSviewer 1.6.18 keyword co-ocurrence analysis of the 50 included studies (scale 0–1; higher values indicate greater conceptual centrality). Growth percentages for emerging factors represent the increase in publications between 2020–2022 y 2023–2025 sun periods. 2023 Projections are estimates based on observed growth trends. Al = Artificial intelligence; IL = Information Literacy. Bibliometric analysis compiled by the authors.

The specific geographical análysis of higher education by región was also analyzed: it show that in Europe it has 26%, Asia 24%, North America 16%, Africa 12%, Latin America 14% and a tie between Oceania and the Middle East with 4%; in relation to the gaps it is evidente that in Sub-Saharan Africa and Central Asia the priority index is critical (See Table 5).

Regarding measurement instruments for university contexts, the IL Doc Model, IL- HUMASS Survey, DigComp 2.1 Adaptation, IL Engineering Scale, and the GAI Critical Literacy Scale were identified. Contexts, reliability, latent factors, and sample size with their units of analysis were also identified for each of the identified instruments.

The predominant methodologies for analysis in higher education identified were: correlational, quasi-experimental, experimental, longitudinal and systematic review designs. Representative studies and their advantages in the university context are also included (See Table 6).

It is considered important to establish the thematic evolution by period, which was divided into three periods: 2015–2018: Foundations of University IL; 2019–2022: University Digitization and 2023–2025: AI and University Critical Thinking. Emerging factors include critical AI literacy with a potential transformational impact, competency verification with a high potential impact, assisted academic writing with high potential, and academic digital collaboration with a medium–high potential (See Table 7).

The quality criteria considered for this review were: University relevance, Methodological rigor, Sample size, Validated instruments and Practical applicability, classified as Excellent, Good, Acceptable or Poor (See Table 8).

A bibliometric analysis of 50 specific articles on research literacy in higher education (2015–2025) reveals a field undergoing rapid consolidation, with an annual growth rate of 26.3%. Institutional factors show the largest average association (average d = 0.83), particularly library-faculty collaboration (d = 0.94) and institutional IL frameworks (d = 0.87). Personal factors show the most consistent associations, led by information literacy (d = 0.82) and research self-efficacy (d = 0.76). Geographically, a Europe-Asia concentration persists (50%) with critical underrepresentation of Sub-Saharan Africa (3.5x) and Central Asia (6.0x). Emerging trends toward critical AI literacy (+410%) and verification skills (+280%) point to a fundamental paradigm shift.

The average methodological quality (77.5%) indicates disciplinary maturity, although longitudinal limitations persist (12% of studies) and require priority attention to understand skill development throughout the university experience.

Figure 2 shows the interconnected dimensions which show that most studies focused on the personal human factor (green cloud), followed by the pedagogical (blue cloud) and to a lesser extent on the institutional (red cloud); however, the three are connected making it impossible to understand them separately as the dimensions influence each other.

**Figure 2.**
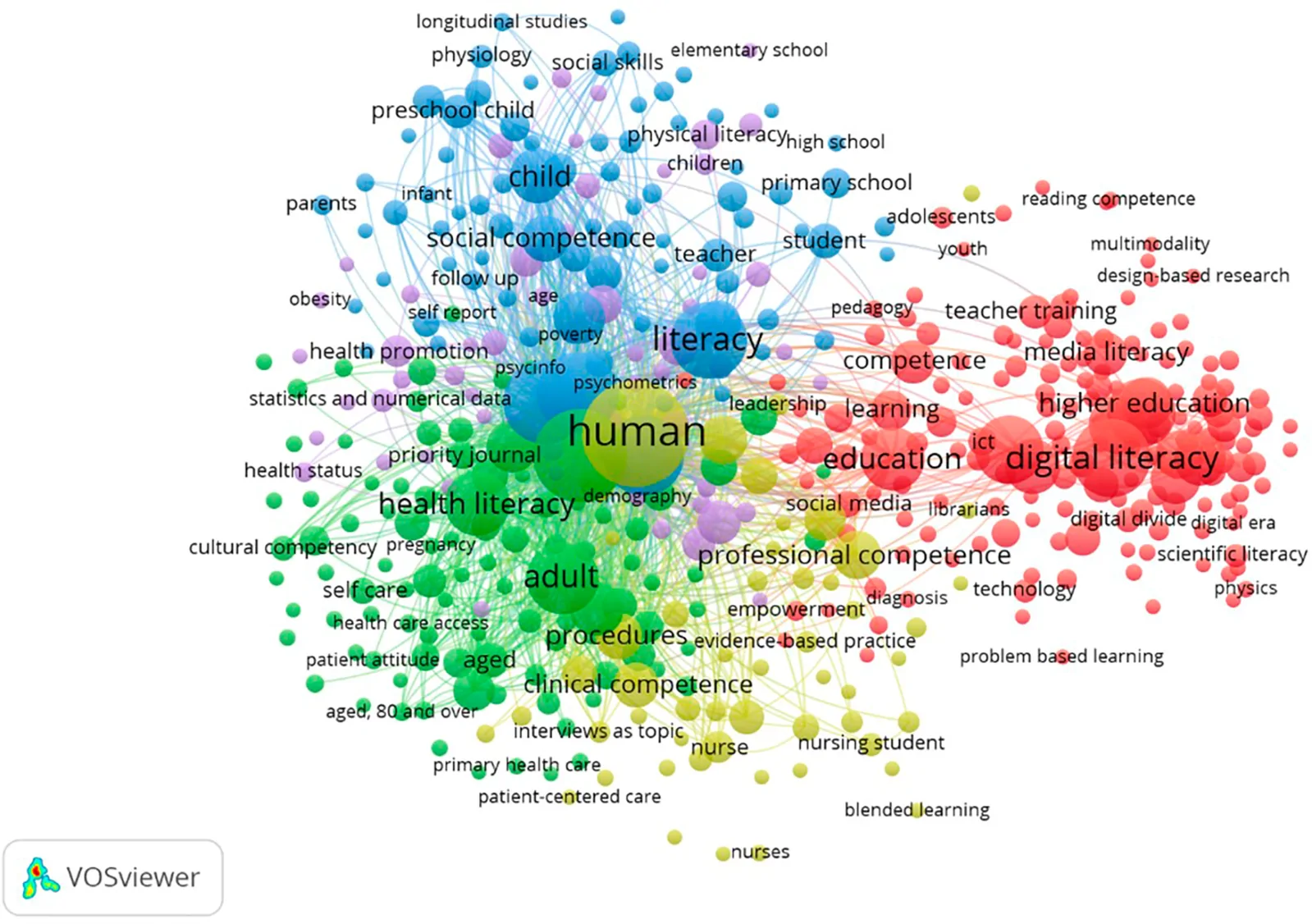
Integrative framework of the three interconnected dimensions of university research literacy: personal, pedagogical and institutional. *Note:* This framework synthesizes findings from the 50 reviewed studies. Circle size reflects the proportion of the 35 studies with extractable quantitative data addressing each dimension: personal (63%, n = 22), pedagogical (23%, n = 8), and institutional (14%, n = 5); the remaining 15 studies inform the framework narratively. Bidirectional arrows indicate that dimensions mutually influence each other and cannot be interpreted in isolation. The directional structure of the model — institutional conditions acting on instructional opportunity, and instructional opportunity acting on individual competence through self-efficacy — is set out in “From a taxonomy to an explanatory model” in the Discussion. Visualization generated with VOSviewer 1.6.18.

The bibliometric analysis reveals that research literacy is a multidimensional competency; strong links with digital and media literacy are identified, indicating that the current university environment demands technological skills to access, evaluate, and produce scientific knowledge. Consequently, this dimension relates to teacher training and the use of ICT, demonstrating that training in digital environments is essential for fostering academic research. Likewise, a professional and applied dimension emerges, particularly in the health sciences, where research becomes a tool for evidence-based decision-making. Furthermore, bibliometrics also suggests that the development of research competencies begins before university: the progression from early childhood education reflects that this is a prolonged educational process requiring sustained pedagogical strategies (See Table 7 in the Annexes).

Finally, active methodologies—such as blended learning and problem-based learning—emerge as key elements in strengthening research skills (See Table 8 in the Annexes).

## Discussion of results

The findings of this systematic review offer a clear yet complex picture of the factors associated with the development of university research literacy; the convergence of evidence from 50 studies — 35 of them contributing pooled effect sizes and 15 contributing narrative evidence only — analyzed from quantitative, qualitative and mixed perspectives, confirms that this is a systemic phenomenon in which the personal, pedagogical, and institutional dimensions do not operate independently but in constant interaction ( Mbandje et al., 2024; Paul et al., 2024).

With regard to personal factors, the data are compelling: information literacy (d = 0.82) and research self-efficacy (d = 0.76) are the variables most consistently associated with competency development across geographic and disciplinary contexts; because these estimates derive largely from cross-sectional designs, they should be interpreted as statistical prediction rather than as evidence of causal influence; large-scale studies such as those by Makinde et al. (2025) and Tachie-Donkor and Ezema (2023) converge on this point with experimental research, a convergence compatible with these factors operating as educational universals, albeit with contextual variations that cannot be ignored. In this regard, the pattern of associations is consistent with self-efficacy acting as a mediator between prior digital competencies and critical thinking; this mediational reading remains a hypothesis to be tested, since no included study estimated a formal mediation model on longitudinal data.

The pedagogical domain, although less represented in the analyzed literature (23% of the studies), presents the highest effect sizes; collaboration between the library and the faculty (d = 0.94) and structured instruction (d = 0.78) emerge as the strategies associated with the largest documented effect sizes. This is no minor finding: it suggests that the interventions associated with the largest gains are those that bring together different institutional actors around a shared goal, combining direct instruction with practical application in specific disciplinary contexts. The evidence from Reeves et al. (2024) and Kautonen and Gasparini (2024) in this regard is particularly consistent.

Institutional factors (14% of the studies) also deserve attention, precisely because, despite being underrepresented in the literature, they show considerable effects. Institutional conceptual frameworks (d = 0.87) and technological resources are associated with larger estimates for personal and pedagogical variables, a pattern consistent with a contextual amplifying function. This implies that, no matter how highly motivated and capable a student may be, without an institutional environment that facilitates access to resources and establishes clear policies for research training, competency development encounters structural barriers that are difficult to overcome.

From the perspective of methodological trends, the predominance of correlational designs (36% of the studies) reflects a consolidation of the field, albeit with evident limitations in establishing causal relationships. The scarcity of longitudinal studies (barely 12%) is one of the most significant gaps identified: understanding how research competencies develop throughout the university experience requires precisely this type of tracking, which cross-sectional designs cannot provide. This gap is even more relevant when considering, as LeMire et al. (2024) point out, that the development of these competencies does not follow predictable linear patterns.

The geographic distribution of the studies reveals another problematic asymmetry. The concentration of research in Europe and Asia (50% of the total) contrasts with the underrepresentation of Sub-Saharan Africa and Latin America, regions that are home to a significant proportion of the world’s university enrollment. This gap is not merely quantitative: it implies that the enabling factors identified in the literature largely reflect educational realities in high-income contexts, whose transferability to other cultural and socioeconomic settings cannot be assumed without specific contextual validation.

One of the most notable transformations emerging from the analysis is the paradigm shift that has been underway since 2023: the emergence of generative artificial intelligence has redefined the very concept of research literacy. Factors such as critical thinking take on new dimensions when applied to the evaluation of AI-generated information ( Arthi & Gandhimathi, 2025), and the exponential growth of studies on critical AI literacy (+410% between 2023 and 2025) suggests that this will be one of the fastest-growing fields in the coming years.

Finally, the methodological tension between quantitative and qualitative approaches should be recognized as productive rather than as a limitation. While quantitative studies tend to operationalize research literacy in terms of measurable competencies, qualitative approaches highlight process-oriented and contextual dimensions that elude standardization. This duality suggests that research literacy is a hybrid construct that requires conceptual frameworks capable of articulating both dimensions, guiding both future research and the design of pedagogical interventions based on robust empirical evidence.

### From a taxonomy to an explanatory model: mechanisms linking the three levels

In its initial formulation, the individual–instructional–institutional framework operated primarily as a device for classifying the literature. Classification, however, does not by itself constitute theory: it states where a factor sits, not why it matters or through what route its influence travels. We therefore restate the framework as an explanatory model that distinguishes four analytically distinct roles — antecedents, mechanisms, outcomes, and moderating or contextual conditions — and specifies the pathways that connect them.

*Antecedents.* Institutional conditions — information-literacy policy frameworks, library staffing and budget, technological infrastructure, and the presence of research training in curricular regulations — are treated as distal antecedents rather than as direct causes of student competence. They function as contextual cues: they signal to academic staff what the institution rewards, resources and expects, and in doing so they determine the opportunity structure within which teaching decisions are made. This reading is consistent with the observation that institutional factors are underrepresented in the corpus (5 of the 35 pooled studies) yet carry among the largest estimates (institutional IL frameworks, d = 0.87): a distal variable that reliably shapes the proximal ones will appear strong precisely because its effect is transmitted rather than direct.

*Mechanisms.* Two proximal mechanisms are proposed to carry the institutional signal into student learning. The first is instructional opportunity: institutional conditions determine whether library–faculty collaboration, structured instruction and active methodologies are actually enacted, and with what frequency, duration and disciplinary embedding. That the pedagogical level is the least represented in the corpus (8 of 35 studies) while yielding the highest single estimate (library–faculty collaboration, d = 0.94) is what this model predicts: instruction is the point at which institutional capacity is converted into student-facing practice. The second mechanism is motivational–regulatory: repeated, scaffolded exposure to authentic research tasks generates mastery experiences that raise research self-efficacy (d = 0.76), which in turn governs the deployment of metacognitive and critical-thinking strategies (d = 0.63 and d = 0.68) during genuine information problems. Self-efficacy is thus positioned as a mediator, not as a parallel predictor.

*Outcomes.* The distal outcome is research literacy understood as enacted competence — the demonstrated ability to identify an informational need, evaluate evidence, synthesise across sources and communicate findings ethically — rather than as self-reported confidence. This distinction is not cosmetic: Santander-Gana et al. (2024) and Chung et al. (2024) both document a gap between perceived and observed competence, which implies that self-perception and performance are distinct outcomes that a single framework must keep apart.

*Moderating and contextual conditions.* Four conditions are proposed to moderate the strength of the pathways above rather than to enter them as main effects: discipline ( Kadiri et al., 2024, report divergent self-efficacy and motivation profiles between health and non-health programmes); academic stage, since LeMire et al. (2024) show that competence does not accumulate linearly across the undergraduate years; prior digital competence, which sets the floor from which instruction can operate (d = 0.71); and the regional resource context, which plausibly explains part of the geographical asymmetry documented in Table 5 and constrains the transferability of estimates obtained in high-income settings.

*Testable propositions.* Stated as an explanatory model, the framework yields propositions that are empirically falsifiable and that the present evidence base cannot yet adjudicate. P1: the association between institutional IL frameworks and student research literacy is fully or partially mediated by instructional opportunity, so that controlling for the presence and intensity of instruction should substantially attenuate the institutional estimate. P2: the association between structured instruction and enacted competence is partially mediated by research self-efficacy. P3: the effect of instructional opportunity on enacted competence is moderated by prior digital competence, being weakest where that floor is lowest. P4: institutional conditions exert no direct effect on student competence once instructional opportunity is modelled. Testing P1–P4 requires multilevel longitudinal designs with students nested within courses and courses within institutions — precisely the design that is scarcest in the present corpus (12% longitudinal) — and constitutes, in our view, the most consequential agenda for the next generation of empirical work in this field.

## Conclusions

This systematic review of 50 studies published between 2015 and 2025 confirms that university research literacy is a multidimensional, dynamic, and deeply contextualized competency; furthermore, personal factors—led by information literacy (d = 0.82) and research self-efficacy (d = 0.76)—show the most consistent evidence across different geographic and disciplinary contexts, supporting their role as the strongest statistical predictors of competency development, without implying causal precedence. At the same time, pedagogical factors—though less prevalent in the literature—exhibit the greatest effects, particularly library-faculty collaboration (d = 0.94), which invites a rethinking of the organization of university educational processes. Institutional factors, for their part, are associated with larger personal and pedagogical estimates — a pattern consistent with an amplifying function — and their underrepresentation in the reviewed literature constitutes in itself a call for future research.

From a methodological standpoint, the predominance of correlational designs and the scarcity of longitudinal studies are the most significant limitations in the field. Without the complete trajectories of competency development that only longitudinal studies can provide, it is difficult to understand how and when the most significant changes in university students’ research training occur. This gap must be addressed as a priority in the coming years.

Another important limitation of the present study is the geographical concentration of the analyzed literature. The fact that Europe and Asia account for 50% of the reviewed studies not only reflects imbalances in global scientific output but also limits the possibility of generalizing the findings to contexts such as Latin America, Africa, or the Middle East, which remain significantly underrepresented. The cross-cultural validity of the identified enabling factors needs to be verified in these settings.

In terms of practical implications, the results of this review point in a consistent direction: the interventions associated with the largest effect sizes are those that combine explicit instruction in information literacy with active methodologies, collaborative work between the library and faculty, and institutional policies that guarantee adequate resources and structured research training programs. For faculty, this involves integrating the development of research competencies into their daily practices; for university administrators, it entails formalizing inter-institutional collaboration and ensuring relevant technological resources. For curriculum designers, it means incorporating these competencies across the board into the curriculum.

Finally, the paradigm shift driven by artificial intelligence in university educational settings cannot be ignored. Twenty-first-century research literacy increasingly includes the ability to critically evaluate information generated by AI systems, verify its reliability, and use it ethically. The 410% increase in studies on critical AI literacy between 2023 and 2025 suggests that this will be a central field of research in the coming years, and that available conceptual frameworks and assessment tools will need to be continuously updated to reflect a rapidly evolving educational reality.

## Data Availability

The data is available at: Figshare. Qualification: Lista de verificación, diagrama de flujo y base de datos PRISMA. DOI: https://doi.org/10.6084/m9.figshare.31872769 [ Paredes (2026)]. License: CC BY 4.0.
