# Supplementary material for: Factors Associated with Research Literacy in University Students: A Systematic Literature Review 2015-2025
Source: F1000Res. 2026 Oct 17;15:1014. Originally published 2026 Jun 25. [Version 2] doi: 10.12688/f1000research.179559.2 (PMC13691356; doi:10.12688/f1000research.179559.2)
Supplement: Peer review history

# Reviewer report: Reviewer response for version 2

**DOI:** 10.5256/f1000research.209431.r531062

**Published:** 2026-10-22

**Authors:** Xuefei Lin

**Affiliations:** 1 College of Mathematical Science, Yangzhou University College of Mathematical Sciences, Yangzhou, Jiangsu, China

Thank you to the author team for their careful revisions. I no longer have any new questions. I think the article can be published.

Are the rationale for, and objectives of, the Systematic Review clearly stated?

Yes

Is the statistical analysis and its interpretation appropriate?

Partly

If this is a Living Systematic Review, is the ‘living’ method appropriate and is the search schedule clearly defined and justified? (‘Living Systematic Review’ or a variation of this term should be included in the title.)

Yes

Are sufficient details of the methods and analysis provided to allow replication by others?

Yes

Are the conclusions drawn adequately supported by the results presented in the review?

Yes

Reviewer Expertise:

Digital Resources and Higher Education, Artificial Intelligence and Higher Education

I confirm that I have read this submission and believe that I have an appropriate level of expertise to confirm that it is of an acceptable scientific standard.

## References

[1] : Factors Associated with Research Literacy in University Students: A Systematic Literature Review 2015-2025. *F1000Research* .2026;15: 10.12688/f1000research.179559.2 10.12688/f1000research.179559.2

# Reviewer report: Reviewer response for version 1

**DOI:** 10.5256/f1000research.198086.r513582

**Published:** 2026-08-27

**Authors:** Murod Ismailov

**Affiliations:** 1 University of Tsukuba, Tsukuba, Japan

Overall, the manuscript addresses a relevant topic and provides a useful synthesis of research literacy in higher education. The organization around personal, pedagogical, and institutional dimensions is potentially valuable, and the review includes a substantial body of literature. I would recommend **minor revision**, mainly to improve methodological transparency, internal consistency, and presentation.

 Suggested minor revision comments to the authors

1. **Please reconcile the review period throughout the manuscript.** The title, abstract, research question, tables, and conclusions describe the review as covering **2015–2025**, whereas the inclusion criteria state that eligible studies were published from **2015 to 2024**. Please clarify whether 2025 publications were formally eligible and revise all relevant sections accordingly.
2. **Please provide greater detail on the database search strategy to improve reproducibility.** Although the manuscript lists the principal keywords and Boolean combinations, it states that the queries were adapted for each database without presenting the complete database-specific search strings. Consider providing the exact Scopus, Web of Science, and ScienceDirect search strategies, together with the final search date, preferably in a supplementary table.
3. **The derivation of the reported effect sizes requires clarification.** Several results are expressed as Cohen's *d* (e.g., information literacy *d* = 0.82 and library–faculty collaboration *d* = 0.94), and these values are central to the conclusions. Please briefly explain how effect sizes from different primary studies were extracted, converted, or synthesized and, if a meta-analytic procedure was performed, provide the relevant statistical methodology and heterogeneity assessment. If these values were taken directly from individual studies rather than pooled, this should be made explicit.
4. **Please clarify and standardize the methodological quality assessment.** The Methods describe four quality criteria and a Yes/No/Partial rating system, while later presentation appears to use additional quality classifications. The manuscript should clearly explain the scoring procedure, how “Partial” ratings were treated, and how disagreements between reviewers were handled. Providing the quality assessment for all included studies, rather than only a representative sample, would also improve transparency.
5. **Some conclusions should be phrased more cautiously given the predominance of correlational evidence.** Terms such as “predictors,” “most effective interventions,” and statements implying that institutional factors “amplify” other effects may suggest causal relationships. This is particularly important because the authors themselves acknowledge the predominance of correlational designs and limited longitudinal evidence. Please distinguish associations from experimentally demonstrated effects throughout the Results, Discussion, and Conclusions.
6. **A careful language and consistency edit would improve readability.** There are several grammatical/typographical issues and occasional incomplete or awkward expressions (for example, “available empirical and evidence” and “coherent conceptual and e dimensions”). Please also standardize terminology such as *research literacy*, *research competencies*, *information literacy*, and *digital literacy* where these concepts are intended to represent distinct constructs.

**Recommendation: Minor revision.** The topic and synthesis are worthwhile, but addressing these points would substantially improve the methodological clarity, reproducibility, and precision of the manuscript.

Are the rationale for, and objectives of, the Systematic Review clearly stated?

Partly

Is the statistical analysis and its interpretation appropriate?

Partly

If this is a Living Systematic Review, is the ‘living’ method appropriate and is the search schedule clearly defined and justified? (‘Living Systematic Review’ or a variation of this term should be included in the title.)

Partly

Are sufficient details of the methods and analysis provided to allow replication by others?

Yes

Are the conclusions drawn adequately supported by the results presented in the review?

Yes

Reviewer Expertise:

Minor revisions only are requested

I confirm that I have read this submission and believe that I have an appropriate level of expertise to confirm that it is of an acceptable scientific standard.

# Reviewer report: Reviewer response for version 1

**DOI:** 10.5256/f1000research.198086.r513587

**Published:** 2026-08-25

**Authors:** Xuefei Lin

**Affiliations:** 1 College of Mathematical Science, Yangzhou University College of Mathematical Sciences, Yangzhou, Jiangsu, China

Dear editor,

 I would like to thank the editor for inviting me to serve as a reviewer and for giving me the opportunity to read this high-quality manuscript. The paper focuses on a topic that is both timely and of potential research value: the various factors influencing the development of research literacy among college students. The paper integrates relevant factors at the individual, instructional, and institutional levels, attempting to construct a comprehensive analytical framework, while also emphasizing the importance of critical AI literacy. This topic is highly relevant to research in the field of higher education and has good potential for publication. However, before formal acceptance and publication, the manuscript still has several methodological and conceptual issues that need to be addressed. The following are specific review comments for the author’s reference.

1. The manuscript is primarily presented as a systematic literature review, yet the results and tables report Cohen’s d, 95% confidence intervals heterogeneity statistics, giving the work features of a metaanalysis. Nevertheless, the statistical procedures used to derive and pool these effect sizes are not described in sufficient detail. The authors should explain how effect sizes were extracted or transformed from correlations, regression coefficients, mean differences, and other reported statistics; whether fixedeffect or randomeffect models were adopted; how effectsize dependency was handled; and whether sensitivity analyses or publication bias assessments were performed. If the evidence base is too heterogeneous to support a formal metaanalysis, the procedures should be framed as a quantitative evidence synthesis, and overstatements regarding the statistical precision of pooled estimates should be avoided.
2. The title and abstract refer to studies published between 2015 and 2025, while the methodology section indicates that studies from 2015 to 2024 were also included. This discrepancy needs to be corrected, and the exact search dates should be reported. The authors should also provide the complete database-specific search strings, the dates on which each database was searched, and the number of records retrieved from each source. This will enhance transparency and reproducibility.
3. The manuscript identifies 50 included studies, but the quantitative synthesis appears to involve 35 studies, which are subsequently categorized into 22 individual studies, 8 educational studies, and 5 institutional studies. This requires clarification, as the sum of these three categories is 35, not 50. The authors should clearly distinguish between the entire body of included evidence and the subset that met the criteria for the quantitative synthesis, and explain the status of the remaining 15 studies. A revised evidence flowchart or summary table would be helpful.
4. The manuscript frequently uses terms such as “promote,” “influence,” “effective,” and “predict,” even though the review includes a significant proportion of correlation and cross-sectional studies. The manuscript itself reports that correlational designs constitute the bulk of the evidence base, with relatively few longitudinal studies. Consequently, the current wording sometimes goes beyond what the underlying study designs can support. When the evidence is correlational, the authors should use more cautious terminology, such as “correlation,” “association,” or “statistical prediction.”
5. The individual&teaching&institutional framework may be useful, but in its current form, it largely serves as a classification of the literature rather than a theoretical explanatory model. The manuscript should clarify the mechanisms linking these three levels. For example, the authors could conceptualize institutional conditions as contextual cues that shape teaching opportunities, which in turn influence students’ individual research capabilities. The revised framework should distinguish between antecedents, mechanisms, outcomes, and possible moderating or contextual conditions. This will make the theoretical contribution more substantial and provide a clearer agenda for future empirical testing.

Are the rationale for, and objectives of, the Systematic Review clearly stated?

Yes

Is the statistical analysis and its interpretation appropriate?

Partly

If this is a Living Systematic Review, is the ‘living’ method appropriate and is the search schedule clearly defined and justified? (‘Living Systematic Review’ or a variation of this term should be included in the title.)

Yes

Are sufficient details of the methods and analysis provided to allow replication by others?

Yes

Are the conclusions drawn adequately supported by the results presented in the review?

Yes

Reviewer Expertise:

Digital Resources and Higher Education, Artificial Intelligence and Higher Education

I confirm that I have read this submission and believe that I have an appropriate level of expertise to confirm that it is of an acceptable scientific standard, however I have significant reservations, as outlined above.
